# Novel microRNA-based assay demonstrates 92% agreement with diagnosis based on clinicopathologic and management data in a cohort of patients with carcinoma of unknown primary

**DOI:** 10.1186/1476-4598-12-57

**Published:** 2013-06-10

**Authors:** George Pentheroudakis, Nicholas Pavlidis, George Fountzilas, Dimitrios Krikelis, Anna Goussia, Aikaterini Stoyianni, Mats Sanden, Brianna St Cyr, Noga Yerushalmi, Hila Benjamin, Eti Meiri, Ayelet Chajut, Shai Rosenwald, Ranit Aharonov, Yael Spector

**Affiliations:** 1 Department of Medical Oncology, Medical School, University of Ioannina, Ioannina, Greece; 2 Clinical Trial and Applied Research in Oncology Group (CARING), University of Ioannina, Ioannina, Greece; 3 Department of Medical Oncology, “Papageorgiou” Hospital, Aristotle University, Thessaloniki, Greece; 4 Department of Pathology, Medical School, University of Ioannina, Loannina, Greece; 5 Rosetta Genomics Inc, 3711 Market St., Suite 740, Philadelphia, PA 19104, USA; 6 Rosetta Genomics Ltd, 10 Plaut St, Rehovot 76706, Israel

**Keywords:** MicroRNA, Carcinoma of Unknown Primary (CUP), Tumor-of-origin, Molecular diagnostics

## Abstract

**Background:**

Cancer of unknown or uncertain primary is a major diagnostic and clinical challenge, since identifying the tissue-of-origin of metastases is crucial for selecting optimal treatment. MicroRNAs are a family of non-coding, regulatory RNA molecules that are tissue-specific, with a great potential to be excellent biomarkers.

**Methods:**

In this study we tested the performance of a microRNA-based assay in formalin-fixed paraffin-embedded samples from 84 CUP patients.

**Results:**

The microRNA based assay agreed with the clinical diagnosis at presentation in 70% of patients; it agreed with the clinical diagnosis obtained after patient management, taking into account response and outcome data, in 89% of patients; it agreed with the final clinical diagnosis reached with supplemental immunohistochemical stains in 92% of patients, indicating a 22% improvement in agreement from diagnosis at presentation to the final clinical diagnosis. In 18 patients the assay disagreed with the presentation diagnosis and was in agreement with the final clinical diagnosis, which may have resulted in the administration of more effective chemotherapy. In three out of four discordant cases in which supplemental IHC was performed, the IHC results validated the assay’s molecular diagnosis.

**Conclusions:**

This novel microRNA-based assay shows high accuracy in identifying the final clinical diagnosis in a real life CUP patient cohort and could be a useful tool to facilitate administration of optimal therapy.

## Background

Cancer of unknown primary (CUP) is defined as the presence of histologically verified metastases without a clinically detectable primary tumor. CUP constitutes 3%-5% of all newly diagnosed cancer cases, and if cancer of uncertain origin is added, the total number increases to 12-15%. Both CUP and cancer of uncertain origin present a diagnostic as well as a management challenge to clinicians. The identification of tumor origin in metastatic patients is crucial for planning patient management and care since many oncologic treatments include targeted therapies shown to be effective against specific cancers [1-5]. Moreover tumor-specific therapies lead to increased survival of patients with advanced cancers of known origin [5,6].

Identifying the origin of a metastasis is a complex diagnostic process that includes patient history and physical examination, computerized tomography of chest and abdominopelvic cavity supplemented by sign/symptom-directed imaging/endoscopic work-up, histomorphologic assessment of the tumor and immunohistochemistry (IHC) testing of different markers. Although novel IHC markers and development of stepwise algorithms have improved IHC accuracy to 60-88%, they are relatively subjective and fail to determine a single tissue of origin in 20-40% of cases [7-9]. Therefore, molecular diagnostic tools have become an important necessary addition, especially for helping to resolve cases of uncertain primary. Currently, assays for molecular profiling of cancers of unknown primary are available using microarrays and quantitative real-time PCR, for messenger RNA (mRNA) [10-12] or microRNA [13] expression. MicroRNAs are particularly suitable as biomarkers for identifying tumor origin as their expression levels and profile reflect tissue origin and tumorigenesis [14-17]. Moreover, microRNAs have been shown to be highly stable in formalin-fixed paraffin-embedded (FFPE) tissue blocks, the most common and readily available specimen type in pathology [18-20]. Profiling microRNA from FFPE tissue has been described to be superior to mRNA profiling, since the latter are prone to extensive degradation in FFPE samples [20,21].

We have recently described the development and validation of a second generation array-based assay, the 64 microRNA assay (Rosetta Cancer Origin Test™), that identifies 42 tumor types from FFPE tumor samples based on the expression levels of 64 microRNAs [13]. This assay is a significant improvement to the previous first-generation qRT-PCR based assay that identified 25 tumor types using 48 microRNAs. The current novel assay is designed to identify tissue of origin of both metastases and primary tumors from biopsy/resection. The assay was validated using an independent cohort of 509 samples of known origin metastases [13].

Earlier studies validating molecular assays for tissue of origin diagnosis used samples from known origins [11,12,17,22,23]. More recently, a number of studies have been performed assessing molecular assays in the relevant clinical setting of real CUP patients [13,24-29]. Here we present the performance of the 64 microRNA assay in a blinded study on a well annotated cohort of real CUP patients presenting metastases in multiple sites and from multiple origins. In this setting of CUP we compare the assay results with the suspected diagnoses of the patients from initial presentation throughout treatment and follow up. We demonstrate the clinical relevance for management decisions following use of the assay.

## Results

**Figure 1:**
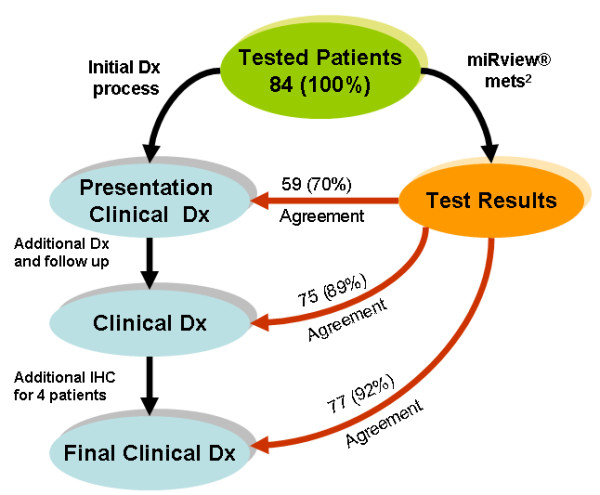
**Diagnostic process and agreement to the 64 microRNA assay.** At the time of referral, each patient was assigned the most likely clinical diagnosis based on all data available at presentation (“Presentation Clinical Diagnosis”). Later on based on additional data gathered during treatment and follow up, each patient was assigned one to three sites of origin (“Clinical Diagnosis”). Finally we performed more IHC tests and arrived at a final diagnosis (“Final Clinical Diagnosis”). For all patients, we independently performed the 64 microRNA assay (assay Results) and compared these to the clinical work-up. The number on each arrow depicts the number of patients (out of 84) for which there was agreement between the test results and the clinical diagnosis, and the number in parenthesis depicts this agreement in percentage. See Methods for more details.

In this study, 93 samples from 92 CUP patients were tested blindly with the 64 microRNA assay. Eight samples failed processing due to inadequate RNA quality. 85 samples from 84 patients were processed successfully (91%) and were assigned assay results. For one patient (12399), two biopsies were made from two different metastasis sites. The two samples yielded the same assay result and hence we report here on 84 results. For each of these 84 patients we obtained both a full clinicopathologic work-up and the result of the 64 microRNA assay. Figure 1 describes the diagnostic process and the agreement with the 64 microRNA assay results. Interestingly, the concordance between the microRNA assay and the clinical diagnosis increased with the progress in clinical work-up, from 70% concordance with the presentation clinical diagnosis to agreement of 89% with the clinical diagnosis during patient management, finally reaching 92% agreement with the final clinical diagnosis, which involved additional IHC studies in four cases (Figure 1). For these concordance studies, the molecular diagnosis took into account the two possible predictions. Thus, the assay results which were obtained based on the initial biopsy/resection were shown to be predictive of the final clinical diagnosis.

For 9 patients (11%), the assay results were discordant with the clinical diagnosis (Figure 1). We selected 7 of those cases for re-evaluation by additional IHC to determine whether the assay may in fact have pointed to an origin missed in the clinical work-up. In addition we selected a case where the assay reported two possible origins, with the second one being concordant with the clinical diagnosis. Sufficient material for re-testing was available in 4 of those 8 cases only (Patient ids: 11805, 12432, 12412, and 11826). Based on the 64 microRNA assay results for these 4 cases, specific IHC markers were chosen and used for IHC staining in order to support or refute the assay results. In 3 out of the 4 re-tested samples, additional IHC work-up resulted in agreement with the 64 microRNA assay results (or in a shift from agreement with the second assay call to agreement with the first assay call). See Table 1 for the description of these 4 cases and the results of the new IHC. In the last part of the results we focus in detail on two of those cases to exemplify the added benefit the 64 microRNA assay may have on patient diagnosis and survival. Following the results of the new IHC performed, the final clinical diagnosis of 3 patients was changed and the concordance of the microRNA-based assay with the final clinical diagnosis reached 92%.

**Table 1:** Immunohistochemical results for 4 cases

**Patient id**	**Original IHC results**	**New IHC results**	**Cancer Origin Test™results**	**Lung Cancer Test™results**	**Clinical Dx prior to new IHC**	**Results (Final Clinical Dx)**
12432	CK7(+), CK8(+), CK19(+), CA125(+), PR (+), CK20(-), CEA(-), TTF1(-), CA19-9(-), VIMENTIN(-)	CK5/6(+, focally), MESOTHELIN(+), CALRETININ(+), EMA(-), LEUM1(-)	Pleural mesothelioma		peritoneal ovarian carcinoma	Peritoneal mesothelioma
12412	none performed	CK 7(+), CK20(-), CA125(+), ER(-), PR(-), CEA(-), p63(-)	Ovarian carcinoma		SCC of the Head & Neck of skin	Ovary peritoneal caricnoma
11826	none performed	CK7 (+), ER(-), PR(-),GCDFP15(-), S-100(-)(rare cells with only weak cytoplasmic immunoreactivity, therefore this staining was considered negative), CEA(-), EMA(+), p63(-), 34E12(+)	Breast carcinoma or SCC of the Anus or Skin		Lung adenocarcinoma or pancreatic adenocarcinoma	Lung adenocarcinoma or pancreatic adenocarcinoma
11805	CK positive and Vimentin negative	TTF1(+), CK7(+, focally), CK20(-), CHROMOGRANIN(+), SYNAPTOPHYSIN(+), CK13(-), CK14(-), CD56(+)	Lung small cell carcinoma or lung adenocarcinoma	Lung small cell carcinoma	Lung adenocarcinoma or pancreatic adenocarcinoma	Lung small cell carcinoma

Table 2 summarizes the statistics of agreement between the final clinical diagnoses and the 64 microRNA assay result, which is either a single origin or two origins. The analysis divides the patients into four groups;

1) Patients for whom the assay agreed with the final clinical Diagnosis but not with the presentation clinical Diagnosis (21.5%).

2) Patients for whom the assay reported one result that agrees with both the presentation clinical Diagnosis and the final clinical Diagnosis (39.5%),

3) Patients for whom the assay reported two results, at least one of which agrees with both the presentation clinical Diagnosis and the final clinical Diagnosis (31%).

4) Patients for whom the assay results did not agree with the final clinical Diagnosis (8%).

**Table 2:** Differential diagnosis of patients

**Patient group #**	**Group description**	**n (%)**	**High confidence of presentation clinical Dx**	**Presentation clinical Dx & microRNA test agreement**	**Final clinical Dx & microRNA test 1st result agreement**	**Final clinical Dx & microRNA test 2nd result agreement**
**1**	**microRNA-based results agree with Final Clinical Dx but not with Presentation Clinical Dx**	**18 (21.5)**	**3/18**	**0/18**	**16/18**	**2/18**
**2**	**microRNA-based test reported a single result which agreed with Final Clinical Dx and with Presentation Clinical Dx**	**33 (39.5)**	**22/33**	**33/33**	**33/33**	**0/33**
**3**	**microRNA-based test reported two results which agreed with Final Clinical Dx and with Presentation Clinical Dx**	**26 (31)**	**13/26**	**26/26**	**25/26**	**1/26**
**4**	**microRNA-based results are not consistent with Clinical Dx**	**7 (8)**	**0/7**	**0/7**	**0/7**	**0/7**

It is important to note that from the 33 patients in the cohort that received two assay answers (group 3), only three patients had their concordant results from the second answer, whereas for 28 patients the agreement was with the first (most probable) answer.

The first group (18 patients) includes patients who would have benefited the most from access to the 64 microRNA assay result, since the assay in those cases was not in agreement with the presentation clinical diagnosis and was in agreement with at least one of the final clinical diagnoses.

### Case report 1 – patient id # 12432

**Figure 2:**
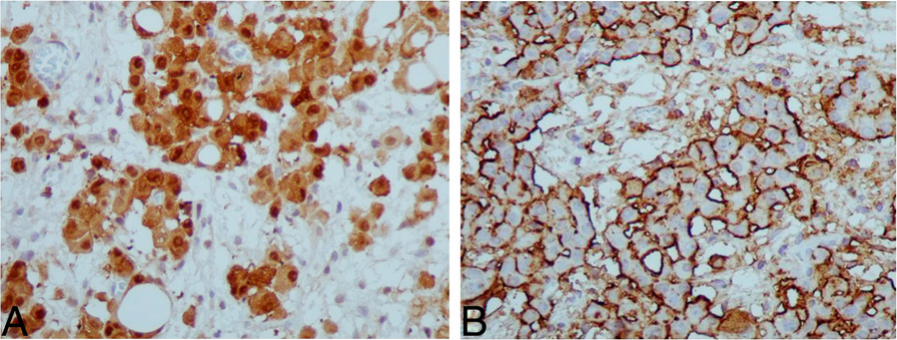
**IHC results for patient # 12432 (Case report 1).** Results of further IHC performed for patient #12432 following the 64 microRNA assay results: **A** Calretinin X400, **B** Mesothelin X400

A 61-year-old female patient, suffering from peritoneal carcinomatosis and ascites, underwent laparoscopic adnexal, ovarian and peritoneal biopsies and was diagnosed with CUP. Ovaries were normal, but the adnexal biopsy disclosed an adenocarcinoma positive for CK7, CK8, CK19, CA125, and PR and negative for CK20, CEA, TTF1, CA19-9 and vimentin. Initial clinical and pathological information suggested a diagnosis of primary peritoneal or ovarian carcinoma. Throughout her care, a potential diagnosis of endometrial cancer was added in the differential diagnosis. The patient did not respond to first line taxane-platinum therapy as anticipated but exhibited a rather indolent disease course, reaching an overall survival of 30 months on 2^nd^ line oral vinorelbine, followed by best supportive care only. The 64 microRNA assay provided a single answer of pleural mesothelioma. Since this result did not agree with the presentation clinical diagnosis, nor did it agree with the clinical diagnoses, additional IHC tests were performed (CK5/6, mesothelin, calretinin, EMA, and LEUM1, see Table 1, Figure 2). Pathologic review of the additional IHC stains agreed with the diagnosis of mesothelioma, although it was still debated whether it was pleural or peritoneal (the 64 microRNA does not differentiate between pleural or peritoneal mesothelioma). The revised diagnosis would have implicated a change of therapy from platinum/taxane to pemetrexed/platinum salts, which could have resulted in improved survival.

### Case report 2 - patient id # 11805

**Figure 3:**
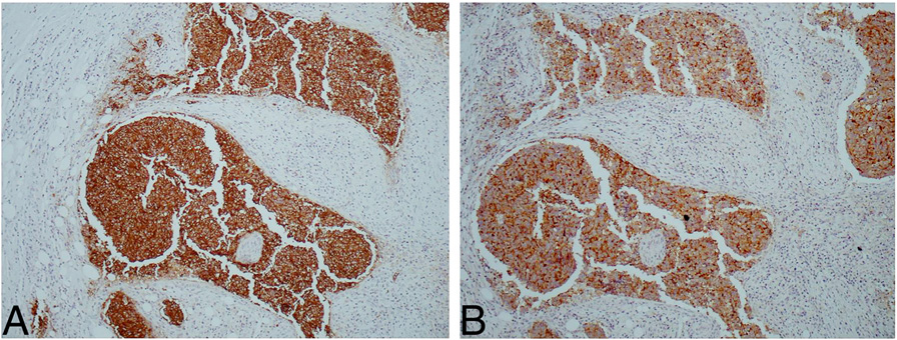
**IHC results for patient # 11805 (Case report 2).** Results of further IHC performed for patient #11805 following the 64 microRNA assay results: **A** Chromogranin X100, **B** Synaptophysin X100

A 60-year-old male presented with a pelvic soft tissue mass, subcutaneous deposits, lung and bony metastases without any evidence of a primary tumor. A subcutaneous deposit was biopsied, disclosing an adenocarcinoma positive for cytokeratins and negative for vimentin. The patient was managed with oxaliplatin + irinotecan for 4 months in the context of a CUP clinical trial, failed to respond and subsequently received paclitaxel/carboplatin combination chemotherapy. This resulted in partial remission of the malignancy and an overall survival of 20 months. The final clinical diagnoses were either lung adenocarcinoma or pancreatic adenocarcinoma. In contrast, the 64 microRNA assay suggested small cell lung carcinoma as the first result, and large cell or adenocarcinoma of the lung as the second result. The biopsied tissue was then analyzed with another microRNA based assay, Rosetta Lung Cancer Test™ [30] and the result supported the first assay answer (the higher confidence result) of small cell lung carcinoma. Additional IHC work up on the archived bioptic material (TTF1, CK7, CK20, chromogranin, synaptophysin, and CD56, see Table 1, Figure 3) resulted in a diagnosis that was in agreement with the 64 microRNA assay first answer of small cell lung carcinoma. This knowledge could have prevented the administration of ineffective oxaliplatin/irinotecan therapy to the patient, and could have resulted in the administration of platinum-based regimens earlier during the course of the disease.

## Discussion

MicroRNAs have been repeatedly shown to be sensitive diagnostic markers to identify tissue of origin [13,17,31]. This potential for tissue of origin identification is evident since microRNAs bear an important role in protein expression regulation and therefore their expression in cells depends on their origin and type.

The importance of correct and rapid diagnosis in CUP patients is critical since the origin of the malignancy dictates the optimal therapeutic treatment for the patient, which could have a positive impact on patient outcomes. Standard diagnosis of CUP patients is based on pathological evaluation supplemented by IHC tests, patient’s history, clinical presentation and relevant imaging tests coupled to serum tumor markers. Nevertheless, the clinician quite often changes the diagnosis of the suspected tissue of origin or additional suspected origins are added to the differential diagnosis, due to the course of the disease, response to therapy and outcome.

In view of the plethora of IHC stains, and the hurdle to determine the most appropriate stains to use, any additional objective data should help physicians to rationalize the diagnostic algorithm. Therefore, molecular diagnostics are high-value clinical tools that can provide key answers to both pathologists and oncologists.

Both mRNA and microRNA expression have been used to develop specific assays and algorithms to determine the tissue of origin in patient samples. MicroRNAs have been shown to be superior to mRNA when using FFPE samples, the routine preservation method for histological evaluation of biopsies and resections. MicroRNAs are more stable than mRNA in FFPE blocks and microRNA extracted from FFPE blocks show similar profiles to microRNA extracted from fresh tissue [17].

An array-based assay utilizing microRNAs (Rosetta Cancer Origin Test™) was therefore developed and validated. The assay is capable of identifying 42 different tumor types using a set of 64 microRNAs. The assay algorithms (KNN classifier and decision tree) were trained using a total of 1282 primary and metastatic samples from known origins. The assay returns either a single tissue of origin or two possible origins. Validation of this 64 microRNA assay on an independent set of 509 samples, from known origins, demonstrated a high level of accuracy; sensitivity for a single answer prediction of 90%, overall sensitivity of 85% and overall specificity of up to 99% [13]. A separate validation study on 52 true CUP cases of CNS origin demonstrated 88% concordance with clinical presentation and pathology [13].

In this study we tested a cohort of real CUP patients presenting metastases in various sites using the 64-microRNA assay, which demonstrated agreement with pathological and clinical information that increased with the clinical course of the patient. In the absence of a clear gold standard diagnosis in true CUP cases it is challenging to assess the agreement between the molecular assay’s results and the clinical diagnosis. The suspected final clinical diagnosis is based on clinicopathologic data, the patient course, response to tissue-specific therapies and survival, and additional IHC tests when needed. The suspected tissue of origin may be revised with time as more data is gathered during patient’s management. The diagnosis based on the clinical and pathological data available at presentation, and without additional data gathered throughout patient management, had only 70% agreement with the assay results. The agreement with the final clinical diagnosis was 92%. Thus, for about 22% of the patients the microRNA assay would have suggested a relevant differential diagnosis that was not considered at presentation and was later considered as one of the final clinical diagnoses gathered throughout patient care. Moreover, in a few cases the 64-microRNA assay triggered the use of additional IHC stains (to test for candidate origins that were not suspected as likely during patient’s diagnosis and treatment), which either validated the molecular diagnosis or contributed to further analysis and correction of the final diagnosis.

Recently, a similar study was published on real CUP patients using an mRNA-based test reaching only 74-77% agreement with clinical and pathological information including IHC, some of which were triggered by the assay results [25].

In order to correctly evaluate the clinical significance of the 64-microRNA assay, we studied specific cases where the added information given to the clinician would have contributed to a revised diagnosis and to a modification of therapy. In fact, for the 18 patients for whom the molecular assay would have resulted in a revision of the presentation clinical diagnosis, different chemotherapy may have been administered in all 18 patients, and the revised therapy would probably have been more active and associated with increased survival in nine patients. Finally, the change in diagnosis would have probably resulted in modification of expensive, targeted therapies in 16 out of 18 patients.

There is controversy whether CUP patients fare better when treated with primary-specific therapy rather than with empiric combination chemotherapy. A recent paper studied CUP patients who received assay-directed site-specific treatment [32]. The results of this research suggest that the survival of patients treated with assay- directed therapy is better than the expected survival for CUP patients described in the literature. Additional studies are required in order to validate these findings.

A clinically challenging question is how to handle cases in which the clinicopathologic and molecular diagnoses disagree. Our stepwise study of molecular diagnosis agreement with the clinical diagnosis at presentation and with the final clinical diagnosis at the conclusion of patient management strongly suggests that the molecular diagnosis is a very powerful tool for correctly identifying the primary.

We therefore conclude that microRNA profiling is indeed a useful adjunct to traditional clinical and pathologic evaluation for CUP cases, when the sample processing yielded a test result. The assay can help by narrowing down the potential diagnostic options and by increasing confidence in a suspected tissue of origin diagnosis, or by suggesting a different origin resulting in more timely administration of optimal therapy.

## Methods

### Patients and sample preparation

93 FFPE CUP blocks were collected retrospectively from 92 patients diagnosed with CUP according to a standardized clinico-pathologic diagnostic algorithm and managed in Hellenic Cooperative Oncology Group (HeCOG) - affiliated centers from 2001 until 2009. Most patients were males (59), and belonged to visceral (29), squamous head neck (18), midline nodal (10), peritoneal carcinomatosis (18) and axillary nodal (9) CUP subgroups. H&E sections and IHC workup from all cases were reviewed centrally by an independent pathologist (AG). Optimal IHC work up was available in 64 cases (median of 8 IHC stains, range 4–12).

Eighty five samples from 84 patients are the subjects of this study as 8 samples had inadequate RNA quality and did not pass the assay’s QA criteria. See Table 3 for patient information. These 84 patients were assigned three diagnoses over time:

**Table 3:** Patient information

***N = 84***		***N***
**Median age**		
**(interquartile range)**	65 (53 – 70)	
**Gender**	Male	45
	Female	39
**Performance status**	0 – 1	61
	≥ 2	23
**Histology**	Adenocarcinoma	50
	Squamous Carcinoma	15
	PDC/UDC	15
	Malignant Neoplasm	4
**Differentiation (grade)**	1	7
	2	33
	3	44
**Clinicopathologic subgroup**	Visceral	29
	Axillary nodal	9
	Peritoneal carcinomatosis	18 (15/3)
	(Serous/Mucinous)	
	Midline nodal	10
	Squamous cervical nodal	18
**Chemotherapy**	Chemo (Yes / No)	63/ 21
	Platinum-based	27
	Platinum + Taxane	23
	Taxane-based	3
	Other	10
**Median PFS (95% CI)**		7 months (4.8 – 9.2)
**Median OS (95% CI)**		12 months (8.7 – 15.3)

1) At the time of referral each patient was assigned the most likely clinical diagnosis based on all data available at presentation (signs, symptoms, metastatic sites, disease bulk, patient’s history, pathological evaluation including IHC testing). We called this ***“presentation clinical diagnosis”***.

2) Later on during the course of the disease each patient was assigned one to three sites of origin. We called these “clinical diagnosis”, as for reaching these ***“clinical diagnoses”*** we took into account additional data gathered during treatment and follow up, such as response to therapies, spatial and temporal pattern of relapse, and survival.

3) Following the molecular diagnosis provided by the 64 microRNA assay, supplemental IHC tests resulted in the ***“final clinical diagnosis”***.

The clinical team and the team performing the assay were blinded to each other regarding the first two diagnoses, and the molecular diagnosis provided by the 64 microRNA assay. Un-blinding was performed only at the time of data analysis to examine agreement between clinical and molecular diagnoses and in order to perform supplemental IHC tests.

### The 64 microRNA assay procedure

The assay was performed on FFPE tissue, and H&E slides were reviewed by a surgical pathologist for suitability regarding tumor cell content, surrounding tissue, amount of necrosis, inflammation, hemorrhage, and fibrosis. The method has been validated for a tumor cell content of at least 50%. When feasible, micro-dissection was performed to increase the tumor cell content to beyond 50% on the basis of tumor size and histologic features. Suitable samples were processed as previously described [13] to generate tissue of origin results. Briefly, total RNA is extracted by using acid phenol–chloroform extraction, and RNA is labeled by ligation of an RNA-linker, p-rCrU-Cy/dye and quantified using custom-designed arrays from Agilent Technologies. Arrays are scanned, and the signal values of 64 assay microRNAs are obtained following normalization, and used as input to the assay’s classifiers. Inadequate RNA quality is defined either as insufficient amount of RNA to run the assay (less than 0.25ug) or, for example, an RNA sample that resulted with less than 65 microRNAs having a signal of at least 500 on the array.

The assay relies on two classifiers to determine the tissue of origin; a K nearest-neighbor (KNN) classifier and a binary decision tree. Each of the two classifiers predicts one of the 42 tumor types or one of seven combined tumor classes (e.g. Adenocarcinoma of Biliary Tract or Pancreas), and assigns a confidence measure to its prediction. The two predictions are then combined into a single predicted tissue origin or two different predictions, based on whether the two classifiers agree, and on their confidence measures. When two predictions are reported, they are ranked by the Positive Predictive Value (PPV) of each answer. When both classifiers exhibit very low confidence in their result, the assay does not generate a result and reports that the microRNA expression pattern of the sample does not match any of the expression patterns in the panel closely enough, which happens in ~2% of cases, and did not happen for any of the cases in this study. See Meiri et. al. for more details on the assay [13].

## Competing interests

Authors affiliated with Rosetta Genomics are or were full-time employees of the company, which stands to gain from the publication of this manuscript. Some of the authors affiliated with Rosetta Genomics hold stocks or stock options of the company.

## Authors’ contributions

GP, NP, GF, DK, AG, AS, MS, BSt.C, NY, HB, EM, AC, SR, RA, YS, GP, NP, AG, AC, RA and YS participated in the design of the study, AS participated in preparing the samples for the study, MS helped with reviewing some of the slides, EM, BSC performed the Rosetta Cancer Origin Test^TM^ on the samples, GP, SR, RA, AC, HB and YS participated in data analysis, GP, NY, YS, HB and RA drafted the manuscript. All authors read and approved the final manuscript
